# Pioneer of Cardiothoracic Surgery - Joaquim Cavalcanti

**DOI:** 10.21470/1678-9741-2022-0372

**Published:** 2023-07-14

**Authors:** Ricardo de Carvalho Lima, Fernando de Souza Cavalcanti, Leonardo Pontual Lima

**Affiliations:** 1 Department of Cardiovascular Surgery, Pronto-Socorro Cardiológico Universitário de Pernambuco (PROCAPE), Faculdade de Ciências Médicas, Universidade de Pernambuco (UPE), Recife, Pernambuco, Brazil; 2 Department of Rheumatology, Universidade Federal de Pernambuco (UFPE), Recife, Pernambuco, Brazil

**Keywords:** History of Medicine, Cardiac Surgery, Biography, Thoracic Surgery, Brazil

## Abstract

Joaquim de Souza Cavalcanti was a pioneer among us - the Brazilian State of Pernambuco and North-Northeast region - in cardiac surgery in its initial phase (Blalock-Taussig surgery and mitral valvulotomy), in thoracic surgery (pneumectomy, lung lobectomy and segmentectomy, lung decortication, and mediastinal tumor resection), and in numerous techniques and operative tactics in general surgery.

## BIOGRAPHY

Joaquim de Souza Cavalcanti (1918-1956) was born on the Penderama sugar cane estate, located in the municipality of Ipojuca (Pernambuco, Brazil), on April 17, 1918. His father, Joaquim do Rego Cavalcanti, was a Major in the Military Police. Maria Augusta Souza do Rego Cavalcanti, his mother, was a housewife. He had nine siblings: Maria Luiza, Tereza, Carmem, Lucila, Isac, Raquel, José Paulo, Antônio, and Célia. His childhood was spent at the sugar cane estate until he was seven years old, when the family moved to Recife, the Pernambuco State capital, in 1926. With his father’s appointment to head the largest prison in Pernambuco (Casa de Detenção), the family lived inside the prison until 1930, when a coup d’état took place in Pernambuco and his father was deposed. His primary school education was at Colégio Oswaldo Cruz, and his secondary school studies at Ginásio Pernambucano, a traditional school highly regarded as a teaching institution. He intended to be a chemist, owing to his strong affinity for the study of chemistry, but in 1932, before taking the university entrance examination, he decided, under the influence of his father, to apply for the medicine course.

In 1948, Cavalcanti married Norma de Almeida Torres, and they had four children: Joaquim, Fernando, Marcos, and Alberto. His wife was a nutritionist, and after his death she preserved his entire medical history for the future, having published two books about him^[1,2]^. Apart from medicine, Cavalcanti was passionate about piano music, having a great admiration for Beethoven and Chopin, and his wife used to play classical pieces for the family. He was one of the founders of the Sociedade Pernambucana de Astrônomos Amadores, having a small telescope at home with which he made apparently unlimited observations of the universe and used to explain the constellations and the universe to his children.

Inheriting a grave condition of atherosclerotic disease from his father, he died prematurely on June 4^th^, 1956, at the age of 38 years, after suffering three acute myocardial infarcts in the short period between 1952 and 1956.

## MEDICAL SCHOOL

Joaquim Cavalcanti entered the Faculdade de Medicina de Recife in 1933, at only 15 years of age, which made it necessary to present a birth certificate with his date of birth changed to 1917 (one year before his actual birth date). In the second year of his medical course, he was accepted in the unit of Prof. Barros Lima, head of the 2^nd^ Surgical Clinic at Hospital Santo Amaro, in Recife. During this period, he wrote a paper on anatomy dealing with testicular migration, presented at the Sociedade de Estudos Anatômicos Benjamin Baptista. In 1936, he published a paper on gallbladder hypogastric fistula in the *Revista de Medicina Acadêmica*. After completing the 4^th^ year of his medical course, he started working at the Hospital Pronto-Socorro do Recife. In his final year of medical school, along with other medical students, he traveled to the city of São Paulo on a cultural mission, taking medical books written by the following doctors from Pernambuco state: Geraldo de Andrade, Aguinaldo Lins, Otávio de Freitas, Barros Lima, and João Alfredo. In the same year, he was president of the Sociedade de Internos dos Hospitais do Recife and President of the 5^th^ Congresso de Estudantes de Medicina.

## MEDICAL CAREER

**Fig. 1:**
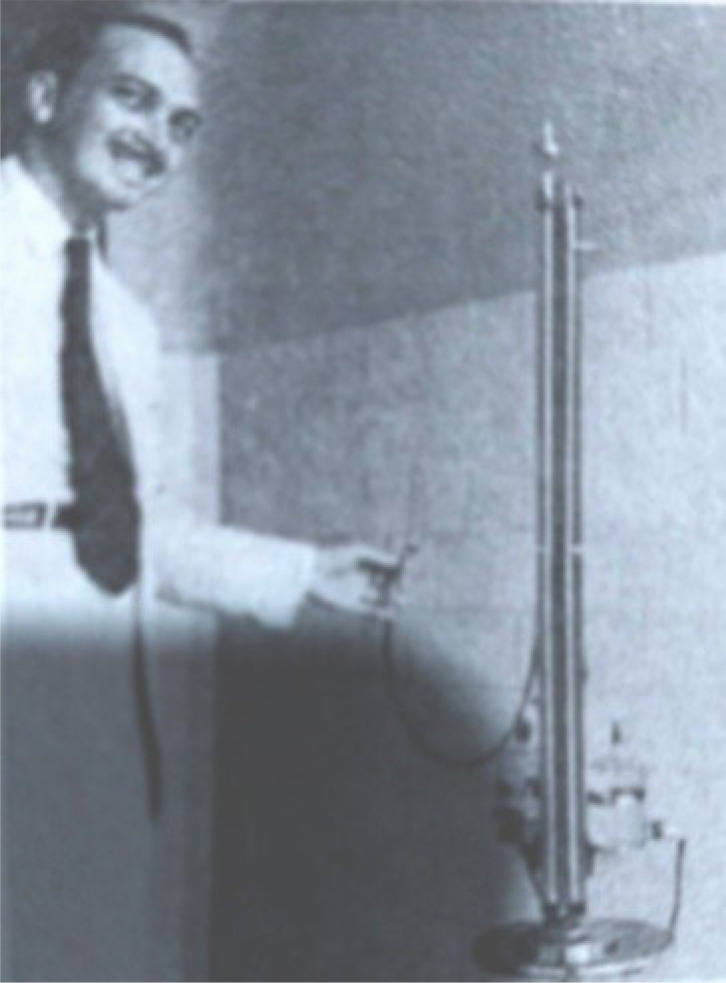
Joaquim Cavalcanti and his device to measure intracavitary pressure.

Joaquim Cavalcanti graduated in Medicine in 1938, at the age of 20 years, as a laureate student, having his father as patron. His father died of an acute myocardial infarction two months after his graduation. Immediately after his graduation, Cavalcanti assumed the position of physician at the Hospital Santo Amaro and of Extranumerary Assistant Professor of the Chair of Pediatric and Orthopedic Surgery at the Hospital da Criança, considered his first step towards a university career. He also assumed the position of general surgeon at Companhia de Seguros Metrópole, at Hospital do Centenário, operating on several patients in the areas of orthopedics and traumatology, gynecology, urology, gastroenterology, and the biliary tract among others. In 1941, Cavalcanti was appointed general surgeon at Hospital Oswaldo Cruz and at the same time, he started to take a keen interest in thoracic surgery, employing the scientific methods of the time with maximum rigor. At this stage, he became particularly interested in the surgical treatment of tuberculosis, an evil that afflicted humanity at that time as there was still no effective clinical treatment for this disease - in 1933, there was no effective surgery for tuberculosis in Recife, except for a few cases of minor phrenic-paralysis surgery and simple costal resections performed by Professor João Alfredo. Cavalcanti’s interest in tuberculosis surgery only increased; as a result, he took a course in physiology at the Liga Pernambucana Contra a Tuberculose and traveled to São Paulo to learn about tuberculosis in the unit of Professor Eurico Bastos and Dr. Eduardo Etzel. Cavalcanti later became an intern at Instituto Clemente Ferreira under the supervision of Dr. Eduardo Etzel, who was the inventor of the rogue surgical instrument for the removal of the periosteum from the rib, considered an invaluable technique throughout the world. Later, in 1941, Cavalcanti was appointed as general surgeon covering the entire state’s hospital network, in particular Hospital Oswaldo Cruz, Hospital de Alienados, and the Leprosário da Mirueira. He was a pioneer in mixed medical techniques in several specialties such as disarticulation with leprosy patients. He also undertook the study of psychiatric disease at the Hospital Psiquiátrico de Tamarineira, where he performed prefrontal lobotomy in patients with mental illnesses - depression, schizophrenia. This was subsequently recognized as being unsuitable for the diseases in question, as Cavalcanti said: “deciding the life of a person who could not even decide about his own life”. He presented a paper at the Congresso de Psiquiatria, Neurologia e Higiene Mental on his experience with spinal cord difficulties caused by war trauma. As his disciple Mauro Arruda succinctly put it: Cavalcanti’s life was “a true via crucis as a surgeon” - owing to the total precariousness of the environment for performing thoracic surgery at Hospital Oswaldo Cruz at that time (1940-1950). The surgical material used was Cavalcanti’s own property, and the lighting for the surgery was handcrafted with the help of a locksmith friend, using car headlights. To cool down the operating room, ice bags were placed on bars minutes before the surgery. That was the way in which thoracic surgeries were performed at that time. In Cavalcanti’s view, surgery was a valuable mean of combating tuberculosis. He also devised a new instrument (Figure 1) for the treatment of tuberculous caverns of the lung, measuring intracavitary pressure, thus promoting continuous aspiration of the lesion.

Pioneer in Thoracic and Cardiovascular Surgery

**Fig. 2:**
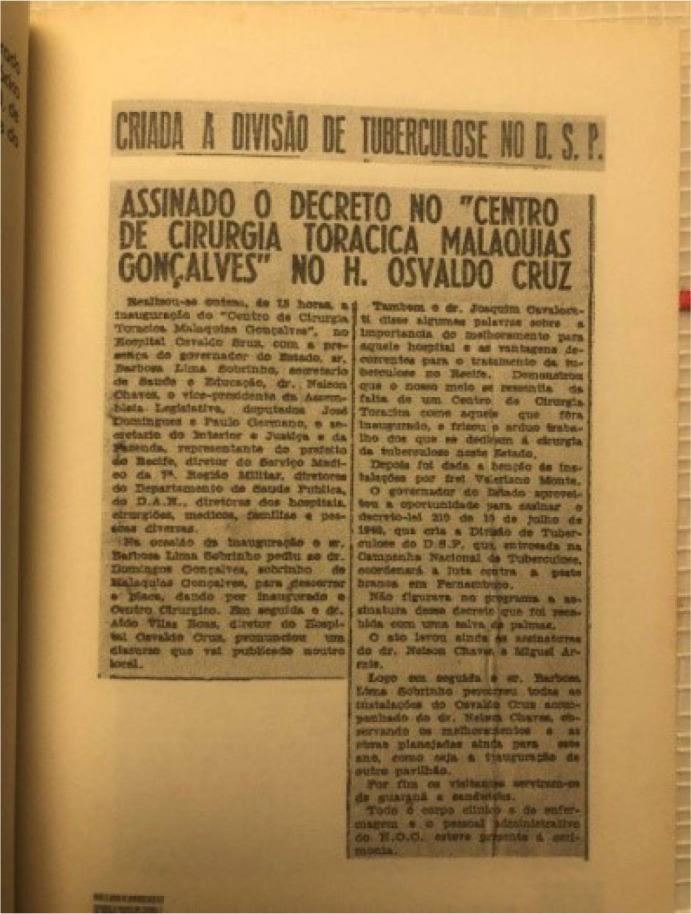
Newspaper report of the time - inauguration of the Centro de Cirurgia Torácica Malaquias Gonçalves.

In 1944, at Hospital Oswaldo Cruz, Joaquim Cavalcanti initiated modern thoracic surgery in Pernambuco State. Until that date, only 148 patients with thoracic pathologies had been operated on at that hospital. Together with wealthy members of civil society in the city of Recife, Cavalcanti started a fundraising campaign to build the first thoracic surgery unit in Northeastern Brazil. The first and symbolic collaboration came from the famous violinist Nair Rotman, who dedicated her inaugural concert to the Department of Pulmonary Tuberculosis Surgery, which would be built at Hospital Oswaldo Cruz. The orchestra was conducted by Maestro Vicente Fittipaldi. The Department of Pulmonary Tuberculosis Surgery, known as Centro de Cirurgia Torácica Malaquias Gonçalves (Figure 2), was inaugurated only four years later, in June 1948, in the presence of the then Governor of the State of Pernambuco, Barbosa Lima Sobrinho. From its opening, it was a hive of surgical activity, becoming the starting point of thoracic surgery in Pernambuco. In the years that followed, 2,034 chest surgeries were performed.

Between 1951 and 1954, Cavalcanti made enormous contributions to Brazilian medicine, performing the first surgery to correct a congenital heart disease and surgery for acquired heart disease (mitral valve repair). In 1951, the first palliative surgery (systemic-pulmonary shunt, Blalock-Taussig surgery) was successfully performed on five children for the treatment of tetralogy of Fallot. This occurred just seven years after the pioneering surgery, carried out by Alfred Blalock and Vivian Thomas, in 1944, in the United States of America. In 1954, also at Hospital Oswaldo Cruz in Recife, Cavalcanti performed a pioneering procedure in the region, namely a digital mitral commissurotomy, having successfully operated on two patients with rheumatic mitral stenosis. This feat was performed in the United States of America for the first time by Harken, in 1948, and by Charles Philamore Bailey, in 1949.

### Academic Life

During his academic life Joaquim Cavalcanti held two full professorships: one in 1942, in the Chair of Operative Technique and Experimental Surgery, and the other in 1945, in Clinical Surgery, with the thesis: Toracoscopia e Pneumolise à Jacobeus, both at the Faculdade de Medicina de Recife. In March 1953, Cavalcanti was appointed Full Professor at the Faculdade de Ciências Médicas de Pernambuco, being replaced after his death by another pioneer of cardiothoracic surgery in Pernambuco and the North-Northeast region of Brazil, Prof. Luiz Tavares da Silva. The Joaquim Cavalcanti Chair was transformed into the Discipline of Thoracic Surgery of the Faculdade de Ciências Médicas, Universidade de Pernambuco. At the present time, this Chair is occupied by Prof. Ricardo de Carvalho Lima, a position obtained through a public examination, held in February 2000 for Full Professor of Thoracic Surgery, Faculdade de Ciências Médicas, Universidade de Pernambuco.

**Fig. 3:**
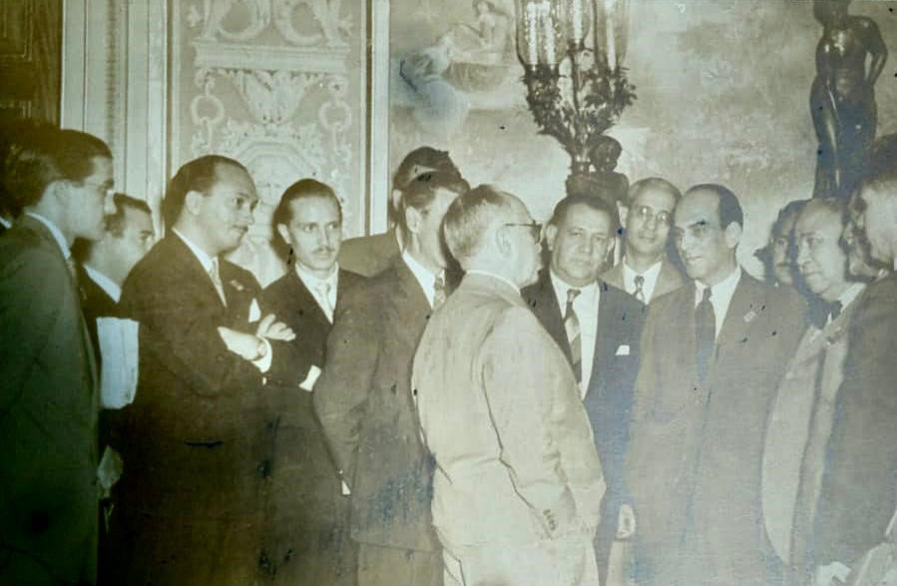
Visit to the President of the Federative Republic of Brazil, Getúlio Vargas.

During his short professional life, Cavalcanti was very active, taking numerous courses. He was an active member of several national and international scientific societies, with internships in São Paulo, London, and the United States of America, with more than 50 works published in Brazilian and American scientific journals, and participation in 27 congresses in Brazil, United States of America, and Europe. He was a member of the editorial board of the *Revista da Associação Médica Brasileira* and 10 other scientific journals in Brazil. Thus, in order to keep his knowledge up-to-date, he subscribed to medical journals in five foreign languages - English, French, German, Spanish, and Italian - and participated in international congresses, presenting his work on several occasions, namely the 33^rd^ Annual Clinical Congress of the American College of Surgeons (1947) and the XIII Annual Meeting of the American College of Chest Physicians (1947) in the United States of America, the I International Congress of the American College of Chest Physicians (1950) in Italy, the VII International Congress of Surgery (1950) in Argentina, and the X Congresso Italiano di Tisiologia (1952) in Rome. In 1954, he participated in the VII Congresso Nacional de Tuberculose and the II Congresso Brasileiro de Doenças Torácicas, held in the city of Rio de Janeiro, when he was part of a delegation received at the Palácio do Catete by the then President of the Republic Getúlio Vargas (Figure 3).

Among us, in Pernambuco State, Cavalcanti was a pioneer in pneumonectomy, pulmonary lobectomy and segmentectomy, pulmonary decortication, resection of mediastinal tumors, cardiac surgery in its initial phase, and numerous operative techniques and tactics in general surgery, performed in Pernambuco State^[3]^ and the North and Northeast of Brazil.

Brazilian North and Northeast surgery will forever be indebted to the work of a true pioneer in the art of thoracic and cardiovascular surgery.

*“Joaquim Cavalcanti always made medicine an ideal. He didn’t derive any great financial rewards from his work, and he didn’t work to obtain such rewards. He worked for the sick, constantly endeavoring to improve his technique”* - Prof. Euclides de Jesus Zerbini

*“Cultured, endowed with an acute intelligence, an excellent presenter, totally devoted to his studies and work - the laborious craft of surgery - for which his capacity was said to be unlimited, he moved through life with agility and effortlessly, without ever abjuring, however, the high ethical standards, both public and private, that characterized his true medical vocation”* - Prof. Jesse Teixeira
